# Angiotensin-(1-7) improves cognitive function and reduces inflammation in mice following mild traumatic brain injury

**DOI:** 10.3389/fnbeh.2022.903980

**Published:** 2022-08-04

**Authors:** Ryan P. Bruhns, Maha Ibrahim Sulaiman, Michael Gaub, Esther H. Bae, Rachel B. Davidson Knapp, Anna R. Larson, Angela Smith, Deziree L. Coleman, William D. Staatz, Alexander J. Sandweiss, Bellal Joseph, Meredith Hay, Tally M. Largent-Milnes, Todd W. Vanderah

**Affiliations:** ^1^Department of Pharmacology, College of Medicine and Health Sciences, University of Arizona, Tucson, AZ, United States; ^2^Department of Surgery, College of Medicine and Health Sciences, University of Arizona, Tucson, AZ, United States; ^3^Department of Physiology, College of Medicine and Health Sciences, University of Arizona, Tucson, AZ, United States

**Keywords:** traumatic brain injury, angiotensin 1-7, Mas receptor, cognitive impairment, pTau

## Abstract

**Introduction:**

Traumatic brain injury (TBI) is a leading cause of disability in the US. Angiotensin 1-7 (Ang-1-7), an endogenous peptide, acts at the G protein coupled MAS1 receptors (MASR) to inhibit inflammatory mediators and decrease reactive oxygen species within the CNS. Few studies have identified whether Ang-(1-7) decreases cognitive impairment following closed TBI. This study examined the therapeutic effect of Ang-(1-7) on secondary injury observed in a murine model of mild TBI (mTBI) in a closed skull, single injury model.

**Materials and methods:**

Male mice (*n* = 108) underwent a closed skull, controlled cortical impact injury. Two hours after injury, mice were administered either Ang-(1-7) (*n* = 12) or vehicle (*n* = 12), continuing through day 5 post-TBI, and tested for cognitive impairment on days 1–5 and 18. pTau, Tau, GFAP, and serum cytokines were measured at multiple time points. Animals were observed daily for cognition and motor coordination *via* novel object recognition. Brain sections were stained and evaluated for neuronal injury.

**Results:**

Administration of Ang-(1-7) daily for 5 days post-mTBI significantly increased cognitive function as compared to saline control-treated animals. Cortical and hippocampal structures showed less damage in the presence of Ang-(1-7), while Ang-(1-7) administration significantly changed the expression of pTau and GFAP in cortical and hippocampal regions as compared to control.

**Discussion:**

These are among the first studies to demonstrate that sustained administration of Ang-(1-7) following a closed-skull, single impact mTBI significantly improves neurologic outcomes, potentially offering a novel therapeutic modality for the prevention of long-term CNS impairment following such injuries.

## Introduction

Traumatic Brain Injury (TBI) is a continuum of neuropathologies resulting from high-energy external force applied to the brain that is subclassified as mild (i.e., concussion) to severe. The Centers for Disease Control highlight TBI morbidity and mortality in the U.S. with an estimated 1.7-million people experiencing TBI annually and 52,000 TBI-related deaths per year (Traumatic Brain Injury/Concussion | Concussion | Traumatic Brain Injury | CDC Injury Center., 2021). Mild TBI (mTBI) is the lowest severity of injury on the TBI spectrum and is often characterized by loss of consciousness lasting <30 min following traumatic, sudden impact to- or rapid acceleration-deceleration of the head (Vos et al., 2002; Menon et al., 2010). Risk factors for mTBI include falls, contact sports, combat, transportation accidents, and physical abuse (Cassidy et al., 2004; Terrio et al., 2009).

The initial *extrinsic* trauma to the brain is accompanied by a secondary *intrinsic* insult mediated by inflammation (Johnson et al., 2013) and increases in reactive nitrogen (i.e., nitric oxide) and oxygen species (Frati et al., 2017). This “second hit” in TBI is a response by the host's immune system to contain local damage; this process causes collateral damage to neurological structures due to its non-specificity (Kulbe and Hall, 2017). Thus, improving TBI outcomes with interventions that target the reversible secondary inflammation and its sequelae are desired (Joseph et al., 2015; Albayram et al., 2017). A recently recognized anti-inflammatory agent is the biologically active heptapeptide Angiotensin-(1-7) [Ang-(1-7)] (Passos-Silva et al., 2013). Angiotensin II is the major product of the renin-angiotensin-aldosterone system (RAAS), responsible for vasoconstriction, fluid retention, and inflammation *via* actions at angiotensin II type 1(AT_1_) and angiotensin II type 2 (AT_2_) receptors (Mehta and Griendling, 2007). Angiotensin-converting enzyme 2 (ACE2) metabolizes angiotensin II into Ang-(1-7). Through actions at the G-protein-coupled Mas receptor (GPCR; MASR), Ang-(1-7) exerts anti-inflammatory (Pörsti et al., 1994), anti-oxidative, and vasodilatory (Jackson et al., 1988; Santos et al., 2003) effects. MASRs are reported throughout the CNS (Jiang et al., 2013) and studies in murine models have shown Ang-(1-7) to confer neuroprotection against CHF-induced cognitive impairment (Hay et al., 2017). Ang-(1-7) also promotes anti-nociception in cancer-induced bone pain (Forte et al., 2016), supporting the role of Ang-(1-7) as an endogenous, physiologic adversary of pathologic inflammation. Our objective was to determine whether Ang-(1-7) confers neuroprotection in mice following closed-skull mTBI. Behavioral, histologic, and biochemical evidence demonstrate an inhibition of mTBI-related secondary injury by Ang-(1-7).

## Materials and methods

### Animals

One hundred and eight male C57/BL6 mice (5.5 weeks, 18–20 g) were housed in a temperature- and humidity-controlled environment on a 12:12 h light:dark cycle. Food and water were available *ad libitum*. All experiments were conducted in accordance with the National Institutes of Health Guide for the Care and Use of Laboratory Animals and approved by The University of Arizona Institutional Animal Care and Use Committee.

### Traumatic brain injury model

A pneumatic impactor delivered a controlled cortical impact (CCI) with desired kinetic parameters (Sandweiss et al., 2017). A closed-skull approach was used for this protocol, whereby skin was incised at midline to expose the calvaria. Parameters were set such that a 7.07 mm^2^ area of cortex located in the left posteromedial parietal lobe (1.5 mm left of sagittal suture, 1–2 mm anterior to lambdoid suture) received a uniform blow (impact: velocity of 4.0 m/s, depth of 1 mm, and dwell time of 500 ms); actual hit velocity for individual animals was recorded immediately post-impact. Mice were anesthetized with 2.5% isoflurane (vol/vol) in an oxygen vehicle (1.5 L/min) before and during impact. Post-impact, mice were monitored for recovery of respiration (time to inspire) and ambulation (time to ambulate). Skin was closed with 5-0 absorbable sutures.

### Drug dosing and testing

Mice received either Ang-(1-7) (1.0 mg/kg total dose, i.p., *n* = 12) or normal saline (0.9%, i.p., 10 ml/kg) vehicle (*n* = 12) 2 h post-TBI, 30 min prior to NOR testing on days 1–5 and day 18, and 30 min prior to sacrifice on day 25. This timeline was chosen to demonstrate a potential maintenance effect on cognitive function in the drug group. Two final doses were administered in order to probe for potential rebound effects. An additional cohort of animals was sacrificed on days 1, 3, and 14 for cortical and hippocampal western blots and ELISAs. An additional three cohorts of mice were administered either normal saline, 0.1 mg/kg Ang-(1-7), or 0.3 mg/kg Ang-(1-7) (i.p., *n* = 10–12) to establish the presence or absence of a dose-response relationship as it pertains to cognitive function. All mouse cohorts were subject to the same cognitive function testing, as described below. Finally, to establish MASR dependence or independence of any observed effect, an additional cohort of mice was subject to pre-treatment with MASR antagonist A779 or saline. In this group, A779 (1 mg/kg, i.p.) or saline was injected i.p. (*n* = 10–12) 30 min prior to receiving 1 mg/kg Ang-(1-7) for 5 consecutive days. These mice were subject to cognitive function testing alongside those having received saline + Ang-(1-7) without antagonistic pre-treatment.

### Cognitive function testing

Novel object recognition (NOR) was employed to test mTBI-induced cognitive impairment (Arenth et al., 2014; Hay et al., 2017; Sandweiss et al., 2017). The apparatus was an evenly illuminated, opaque Plexiglas box (12 cm^3^ 3 × 3 grid on the floor) inside an isolated observation room. A video camera (Canon) acquired an aerial view of mouse exploration/behavior. The time the mouse spent interacting with the objects of the test was tracked offline by three blinded observers both manually and in an objective manner using AnyMaze software; outcome measures in AnyMaze included time with familiar, time with novel, latency to approach, time spent moving, total distance traveled, speed, and number of objects approaches for familiar and novel objects. Objects varied in shape, color, and size. To eliminate olfactory cues, the chamber and objects were cleaned between each mouse and trial. Exploratory behavior was defined as the mouse directing its nose toward the object at a distance of ≤ 2 cm (Hay et al., 2017). The positions of the objects in the test phases, and the objects used as novel or familiar, were counterbalanced between the two groups of mice. Novel object recognition uses three phases of exploration (habituation, familiarization, and testing) to assess an animal's memory in the absence of positive or negative reinforcers.

We employed five trials, as follows:

*Trial 1: Habituation*—The animal is removed from its home enclosure and allowed to freely roam the empty arena (without object present) for 5 min.

*Trial 2: Familiarization*—Three identical objects are placed in their respective crosshairs at opposing corners of the arena. The mouse is re-introduced to the arena and allowed to explore/interact with these identical objects for 5 min.

*Trial 3: Familiarization 2*—Repetition of Trial 2.

*Trial 4: Familiarization with Baseline Collection (Recorded)*—Identical to Trial 2; however, this trial is filmed/recorded in order to collect baseline time measurements and movement data.

*Trial 5: Testing (Recorded)*—Two identical objects are placed in their respective corners; however, the third object is replaced with a single novel object in another corner of the arena. The animal is then allowed to freely roam the arena (as in prior trials) for 5 min. This trial is recorded, and the one from which NOR data are derived.

Of note, familiar objects must always be the same objects for all testing days for a given mouse. For example, if animal 1 begins with three dragons as familiar objects, the familiar objects on all subsequent days must also be dragons. The novel object, however, may never be repeated.

Using multiple measures, the basis for NOR is that “The preference for a novel object means that presentation of the familiar object exists in an animal's memory (Ennaceur 2010).” The recognition of novelty requires more cognitive skills from the subject relative to tasks measuring exploration of novel environments or a single novel object (Silvers et al., 2007). Multiple outcomes can be calculated, including the Discrimination Index (time with novel-time with familiar)/(time with novel + time with familiar), Recognition Index (time with novel)/(time with novel + time with familiar) which corresponds to retention, and Global Index of Habituation (total time spent in exploring each objects during the familiarization phase as compared to the total time spent with each object in the test phase). Thus, an NOR ratio > 0.5 would indicate that (1) cognitive discrimination is intact, (2) retention of familiar object is intact, and/or (3) exploratory behaviors are increased. It is important to note that distance traveled in the context of the above is not altered such that the effect of mTBI on only discrimination and retention can be examined. Discrimination indices (DI) were calculated with a score closer to 1 indicating more time spent with the novel object, whereas scores closer to 0 indicated a preference for familiar objects. To determine if the DI reflected variability in hit parameters over time, correlation analyses were conducted using a simple linear regression.

### Serum collection, cytokine screen, tissue perfusion, and harvesting

Mice were anesthetized with ketamine 80 mg/kg/xylazine 12 mg/kg, i.p. Whole blood samples were acquired *via* a transphrenic approach, allowed to coagulate (30 min, room temperature) and centrifuged (1,000 rpm/4°C) for 20-min. Mice were then immediately perfused with 10 mL of iced 0.1 M PBS. Brains were immediately dissected and blocked in a matrix, taking 3 mm slices from the transverse cerebral fissure ending anterior to the hippocampus. Left cerebral cortex in the region of the injury and *bilateral* hippocampi were carefully dissected in chilled 0.1 M PBS. Serum and tissue samples were snap frozen in liquid nitrogen and stored at −80°C. Cytokine levels were assessed using a commercial kit according to manufacturer's instructions (Invitrogen; SABiosciences, Valencia, CA, USA).

### Westerns

Levels of glial fibrillary acidic protein (GFAP), total Tau, and phosphorylated Tau (pTau) proteins were analyzed by western blot. Tissue samples were homogenized in a Tris-buffered-saline lysis with protease and phosphatase inhibitor cocktail through sonication on ice. Following BCA protein quantification, 15 μg of total protein from each sample was loaded on 10% SDS-polyacrylamide gel wells (TGX Criterion XT; BioRad, Hercules, CA), transferred to polyvinylidene difluoride membrane (BioRad), and blocked with 5% bovine serum albumin in Tris-buffered-saline containing 0.05% (vol/vol) Tween-20 for 1 h at room temperature. Membranes were incubated with primary antibody in 3% BSA/TBST at 4°C overnight. Antibodies and dilutions used rabbit monoclonal anti-GFAP (Abcam ab68428; 1:3,000), rabbit monoclonal anti-Tau (phospho S396, Abcam ab109390; 1:100,000), mouse monoclonal anti-Tau (TAU-5, Abcam ab80579; 1:20,000), mouse monoclonal anti-β-actin (Cell Signaling 7076S; 1:50,000), and mouse monoclonal anti-α-Tubulin (Cell Signaling 3873S; 1:50,000). Blots were incubated in secondary antibody in 3% BSA/TBST for 1 h at room temperature (Cell Signaling 7074 anti-rabbit IgG HRP-Linked, 1:25,000 for GFAP, 1:100,000 for pTau; Cell Signaling 7076 anti-mouse IgG HRP-Linked, 1:100,000 for total Tau, 1:50,000 for β-actin, 1:100,000 for α-Tubulin). Membranes were then incubated in enhanced chemiluminescence solution (Clarity ECL Substrate, BioRad) (5 min) and developed using GeneMate BlueLite Autorad films (BioExpress, Kaysville, UT). Scanned images were processed using Adobe Photoshop (San Jose, CA) and computational analysis of band densities carried out using ImageJ software (NIH). Data were normalized to either β-actin or α-tubulin and reported as fold-change over untreated control.

### H&E staining

Animals were sacrificed at days 1, 3, 7, and 14 post-mTBI. Sectioned slices (40 um) were stained with hematoxylin & eosin (H&E) using the Thermo Scientific Rapid-Chrome Frozen Section Staining Kit (#99-900-01, and lot #413385). Directly mounted brain sections followed this protocol: hematoxylin (10 s), 5 rinses in D.I. H_2_O, dip in bluing agent, 5 dips in 95% alcohol, dip in eosin-y, and 5 dips in each of the following solutions: 95% alcohol, 100% alcohol, 100% alcohol, and 2 dips in xylene. For free-floating sections in 30% sucrose in 1x-PBS, 5 min in hematoxylin was used. Images were taken at 4×, 10×, and 20× magnification for assessment of widespread effects on the cortex and hippocampus and closer examination of the injury site, respectively. Image cell counts were quantified using NIH-provided ImageJ software. All images were converted to 16-bit grayscale, with the upper threshold set at 20%. The dimensions were set to 4.5 × 4.5 mm on the area of cortical injury, with circularity adjusted to 0.5–1 to recognize cell structure (a value of 1 indicates a perfect circle) for counting cortical and hippocampal neurons.

### Statistical analysis

Serum protein levels were analyzed by one-way analysis of variance (ANOVA; *post-hoc* Student-Newman-Keuls). NOR time courses were used to create an area-under-the-curve which was analyzed by repeated measure two-way ANOVA with Tukey *post-hoc* analysis (three separate observers were employed, all of whom were blind to treatments). Times recorded from all observers were pooled and averaged. The data were plotted in GraphPad Prism 7 (GraphPad Software, La Jolla, CA) and represent the mean ± SEM with statistical significance represented by ^*^p < 0.05. Group sizes were determined using G^*^Power analysis. A conventional likelihood level β = 0.80 and a conventional significance level α = 0.05 are assumed.

## Results

### TBI in a closed head, closed skull model induces variable levels of cognitive decline

**Figure 1:**
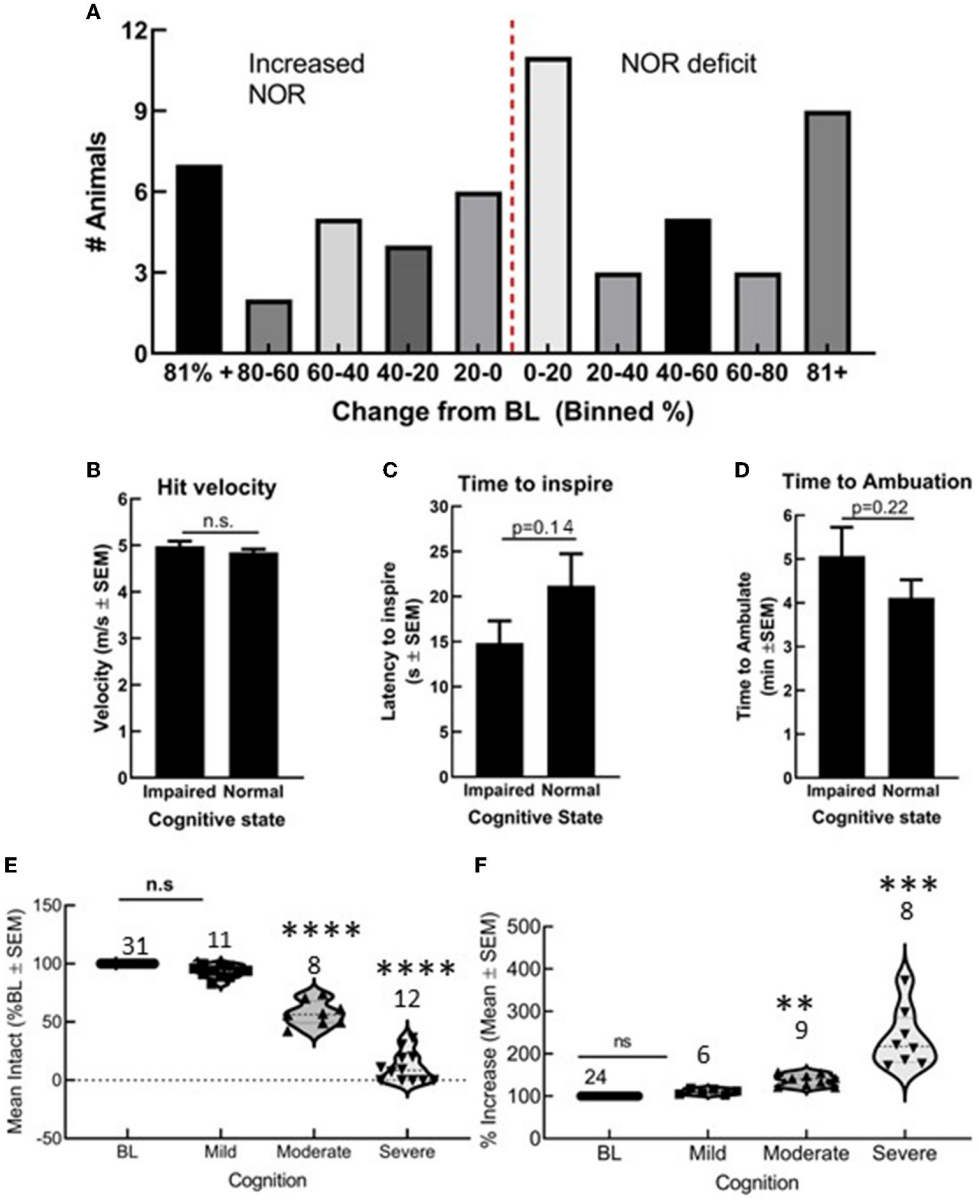
Closed skull traumatic brain injury (TBI) induced variable cognitive outcomes as determined by novel object recognition (NOR). **(A)** Number of animals grouped by 20% bins showing both cognitive decline and cognitive improvement. Differences in **(B)** hit velocity, **(C)** time to inspire, and **(D)** time to ambulate port impact were not different between mice with cognitive impairment or normal cognition. **(E,F)** Animals with a mild TBI (<20% decline or impairment) performed similar to uninjured animals in NOR, whereas moderate and severe outcomes were statistically different from baseline. Statistically significant difference (**p < 0.01, ***P < 0.001, and ****P < 0.0001), *n* = 55 mice.

Fifty-five C57blk/6J male mice received a preprogramed TBI. Of these, 31 showed a reduction in DI values, whereas 24 showed either no change or an increase in DI values (Figure 1). Differences in actual hit velocity (Figure 1B), latency to inspire (Figure 1C), and time to ambulate (Figure 1D) were statistically similar between mice showing decline, vs. stable or improved ability to perform in the NOR task. Grouping post-TBI DI values by percentages suggests multiple types of TBI induced by this model: mild (<20% impairment/improvement), moderate (20–60% impairment or improvement), and severe (>60% decline or improvement) (Figure 1A) that corresponded to significant differences in DI in the moderate range (Figures 1E,F, ^**^p < 0.01). All subsequent assays were performed in animals with a mild/moderate TBI (mTBI).

### Ang-(1-7) prevents post-mTBI cognitive dysfunction

**Figure 2:**
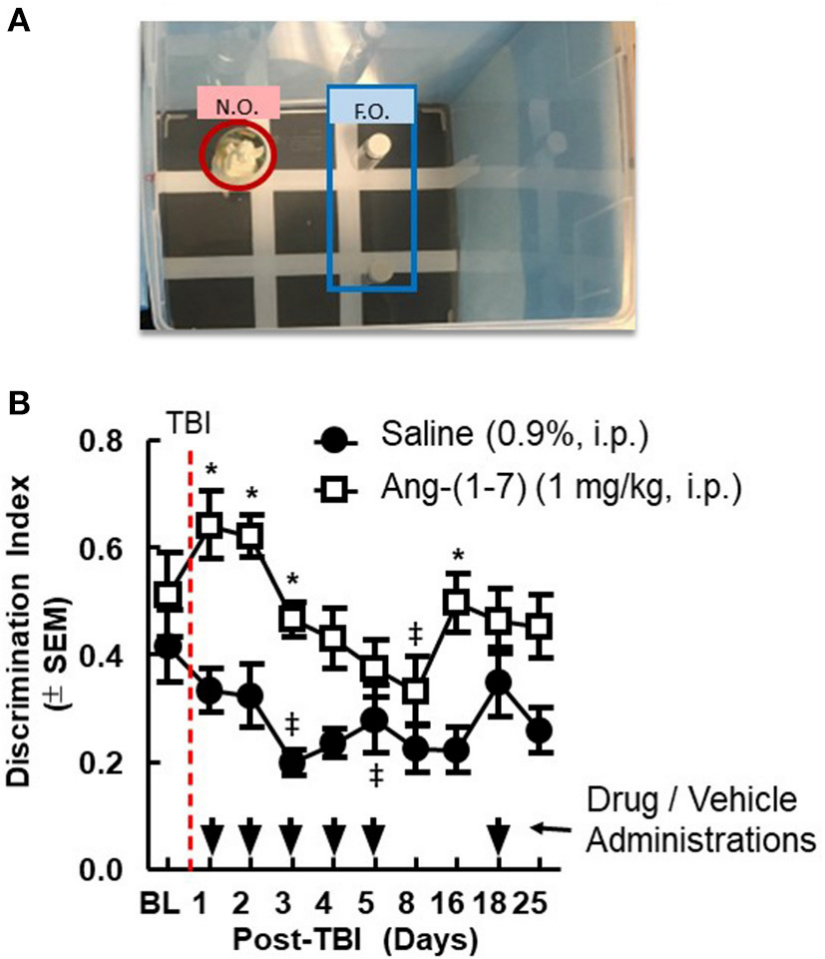
Cognitive deficits after closed-skull TBI are mitigated by Ang-(1-7). **(A)** Diagram of Novel Object Recognition set up. **(B)** Discrimination Ratio as a function of time demonstrates relative maintenance of cognitive function in mice treated with Ang-(1-7) as opposed to normal saline alone. Time of TBI infliction is denoted by the vertical dashed line. Primary administration of either drug or saline vehicle took place 2 h post-TBI. All subsequent drug and saline injections took place 30 min prior to NOR assays conducted on days 2–16. repeated measures ANOVA (with Tukey correction) was employed to analyze main effect of the drug intervention on DR, followed by Tukey Range test where appropriate. Twenty-four male mice 12 mice in each group, statistically significant (*p < 0.05) difference between treatment groups; Statistically significant (^‡^*p* < 0.05) difference from baseline within the group.

Pre-mTBI novel object recognition (NOR) values demonstrated no significant difference between treatment groups (^*^p > 0.05; Figure 2). mTBI significantly reduced DI on days 1, 2, 3, and 16 in saline-treated mice (saline BL to Day 3, *p* < 0.05); Ang-(1-7)-treated mice maintained higher DI values for the duration of the experiment relative to saline control, which aligned with pre-mTBI values. In separate behavioral assays performed on sham mTBI mice, no difference from baseline NOR was observed in mice treated with Ang-(1-7).

### Ang-(1-7)-mediated improvement in cognitive function does not exhibit dose dependence

As in the aforementioned cohorts, pre-injury DI did not differ significantly between groups. mTBI again significantly reduced NOR in most injured mice as evidenced by reduced DI post-injury (Supplementary Figure 1). Ang-(1-7) administration at both 0.1 and 0.3 mg/kg significantly improved NOR in mice having shown initial cognitive deficits post-mTBI (^*^p < 0.05; Supplementary Figure 1). However, DI did not differ significantly between 0.1 and 0.3 mg/kg Ang-(1-7) doses in these initially impaired mice.

### Ang-(1-7)-mediated cognitive improvement is attenuated in the setting of MASR antagonist pre-treatment

**Figure 3:**
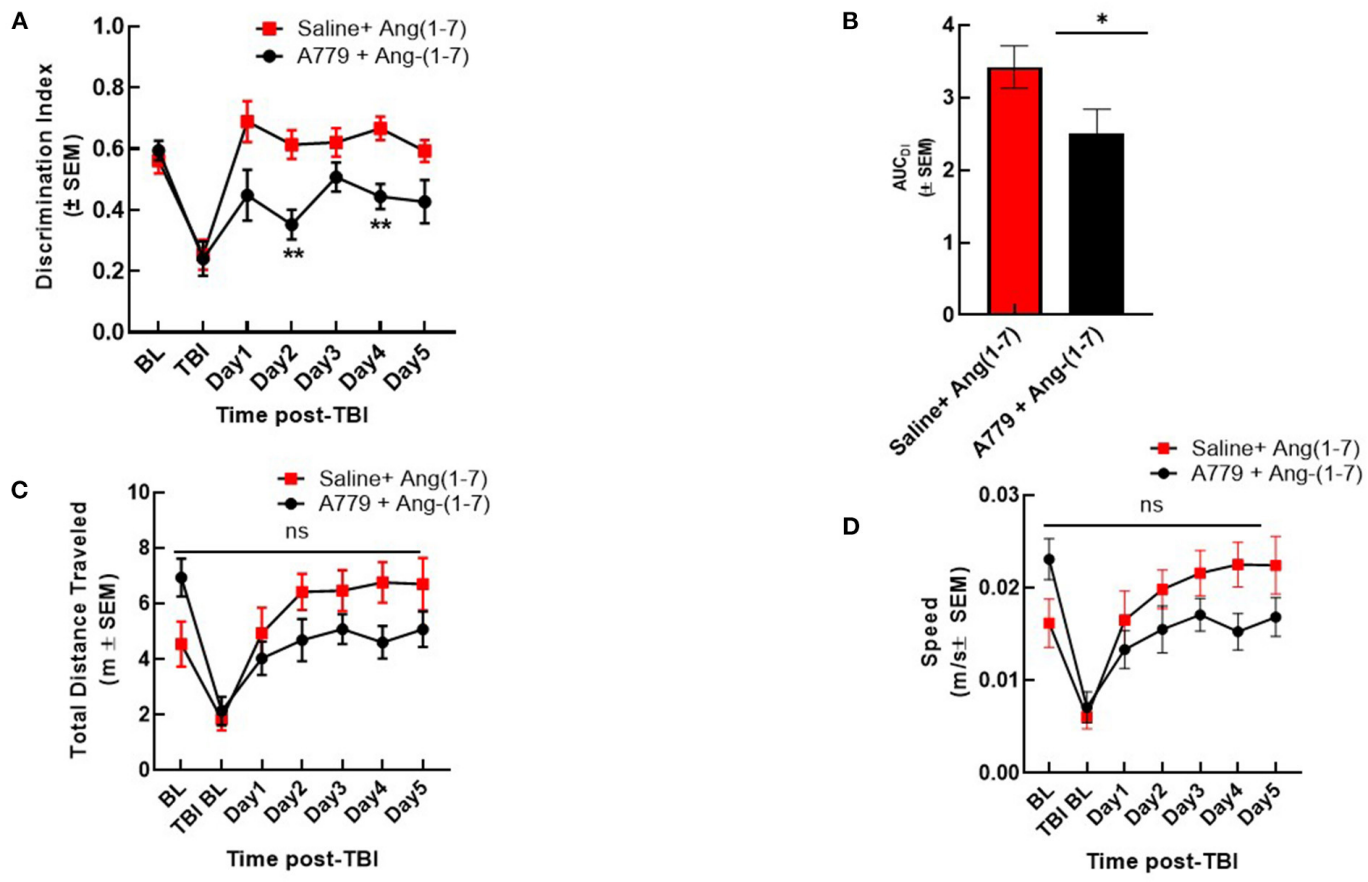
A779 MASR antagonist pre-treatment attenuated Ang-(1-7)-mediated cognitive improvement. **(A)** The NOR discrimination index (DI) was statistically lower in A779 (1 mg/kg, i.p.)-pretreated animals vs. saline on days 2 and 4 of treatment post TBI (*p < 0.05). **(B)** This was confirmed after calculation of the Area under the curve (AUC) to account for differences over time. **(C,D)** Showed no differences in total distance moved or speed were observed (*P* > 0.05). Repeated measures ANOVA (with Tukey correction) was employed. *N* = 12 mice in each group. **P < 0.01, ns; no significant.

To determine if Ang-(1-7) effects were mediated by MASR, a selective antagonist, A779 (1 mg/kg, i.p.) was administered 30 min prior to Ang-(1-7) (Figure 3). Pre-injury DI was again shown not to be significantly different between groups receiving either pre-treatment saline or A779 with Ang-(1-7). mTBI again precipitated significant reduction in DI relative to baseline in both groups (Figure 3A). In mice pre-treated with A779, DI was significantly reduced relative to that of saline-pretreated animals on days 2 and 4 post-mTBI (Figures 3A,B). This deleterious effect of A779 was demonstrated despite subsequent Ang-(1-7) administration in injured mice. Pre-treatment with MASR antagonist A779 did not impair the ability of mice to move, i.e., no difference in total distance moved or speed was observed (Figures 3C,D)

### Ang-(1-7) attenuates neuronal loss in the cortex post-mTBI

**Figure 4:**
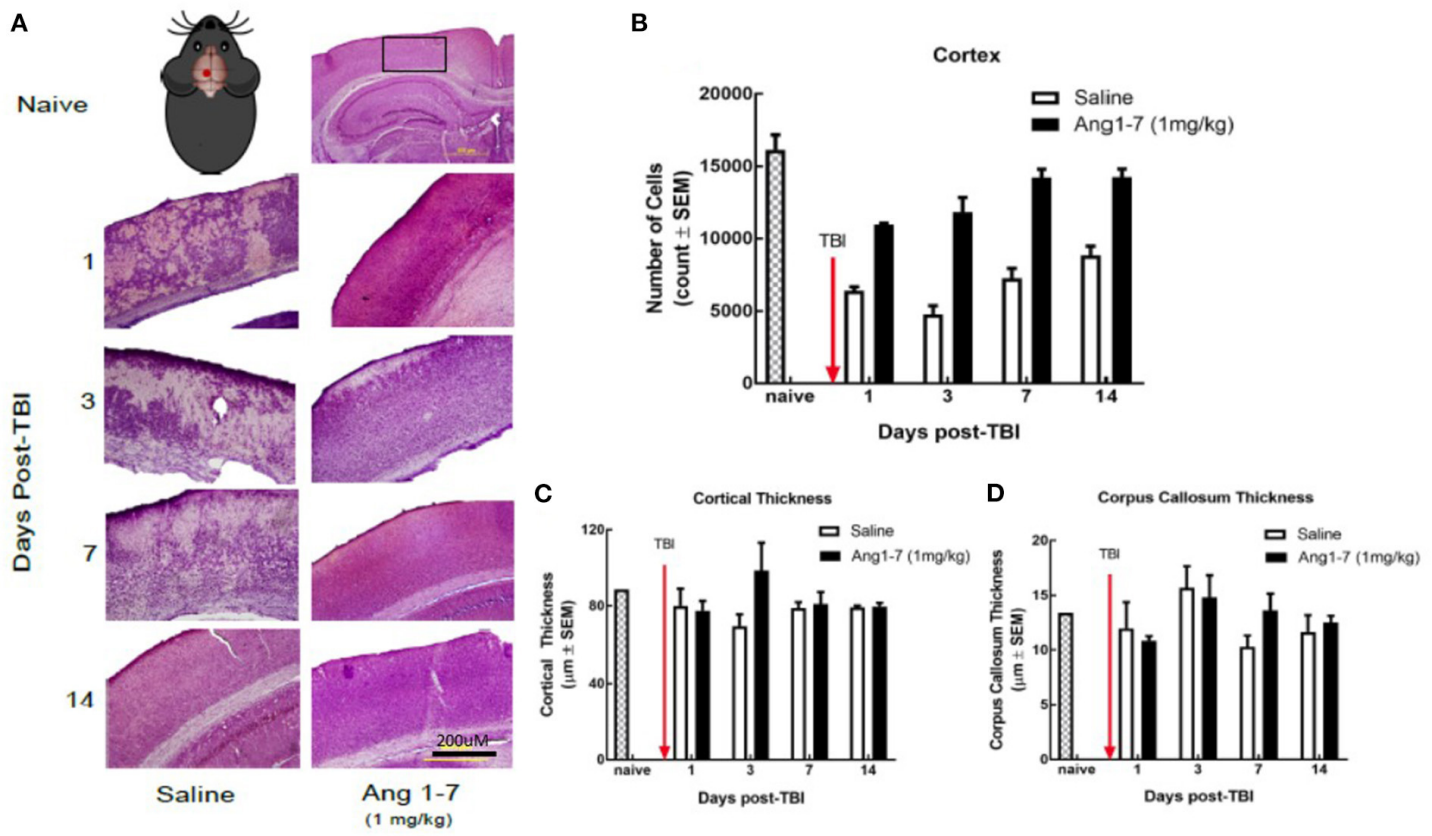
Histology of cerebral cortex after mTBI and Ang1-7 intervention **(A)** HandE staining of cortex for naïve, saline (*N* = 4), and Ang-(1-7) (*N* = 4) treated groups days 1, 3, 7, 14 post-TBI. **(B)** Mouse picture gives location of the single mTBI to the closed skull just above the left parietal lobe. Ang-(1-7) (1 mg/kg, i.p./day on days 1–5) treated mice have significantly higher cortical cell count compared to the saline-treated mice (days 1–5, i.p. *p* < 0.03). **(C,D)** No significant difference was found for cortical thickness and corpus callosum thickness between Ang-(1-7) (1 mg/kg, i.p./day on days 1–5) treated mice and saline treated mice.

Using H&E staining, the effects of Ang-(1-7) on CNS structure was assessed following mTBI. The number of cortical pyramidal neurons was significantly reduced in the saline-treated mice on days 1, 3, 7, and 14 following mTBI (Figures 4A, 2B). Ang-(1-7)-treated mice showed significantly higher cell counts in the cortex compared to that of saline-treated mice on days 1, 3, 7, and 14 post-mTBI (Figure 4B). Ang-(1-7)-treated mice were shown to maintain naïve cell counts on days 7–14 post-mTBI. Both saline-treated and Ang-(1-7) groups maintained stable/upward-trending cell count for the duration of the experiment, except for day 3 in the saline-treated group.

Previous research indicates that mTBI disrupts white matter integrity (Arenth et al., 2014); we measured the thickness of the corpus callosum, as well as overall thickness of the cortex for both (Figures 2C,D). The present study found no significant difference in callosal or cortical thickness between saline and Ang-(1-7) treatment groups (Figures 4C,D).

### Ang-(1-7) reduces neuronal loss in the hippocampus post-mTBI

**Figure 5:**
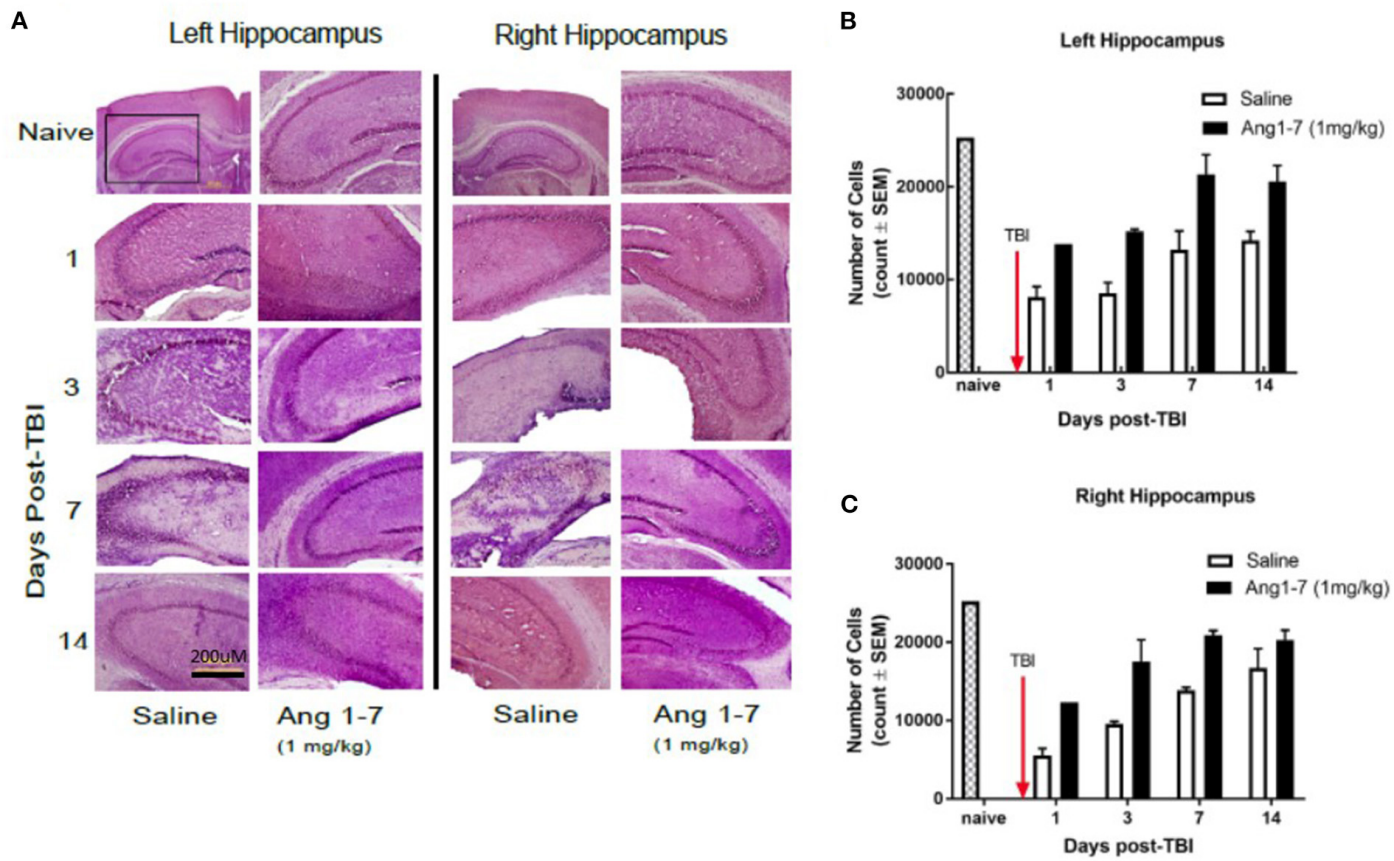
Histology of hippocampus after mTBI and Ang1-7 intervention **(A)** HandE staining of left (ipsilateral) hippocampus and right (contralateral) hippocampus for naïve, saline (*N* = 4), and Ang-(1-7) (*N* = 4) (1 mg/kg, i.p./day on days 1–5) treated groups on days 1, 3, 7, and 14 post-TBI. **(B)** Left hippocampus shows Ang-(1-7) treated mice maintain higher hippocampal cell counts in the CA3 region compared to the saline treated mice on days 1, 3, 7, 14 post-TBI. **(C)** Right hippocampus shows Ang-(1-7) treated mice maintain higher hippocampal cell counts in the CA3 region compared to saline treated mice on days 1 and 3 post-TBI.

The number of hippocampal neurons in the CA3 region was measured for both ipsilateral and contralateral regions with respect to cortical impact. Cell count in the CA3 region of both ipsilateral (left) and contralateral (right) sides was significantly reduced in the saline-treated group on days 1, 3, 7, and 14 following TBI (Figures 5A–C). Ang-(1-7)-treated mice showed a greater ipsilateral cell count as compared to the saline-treated group on days 1, 3, 7, and 14 post-TBI (Figure 5B); similar findings were observed in the contralateral hippocampus (Figure 5C).

### Ang-(1-7) modulates ratio of Tau protein phosphorylation in cortex and hippocampus following mTBI

**Figure 6:**
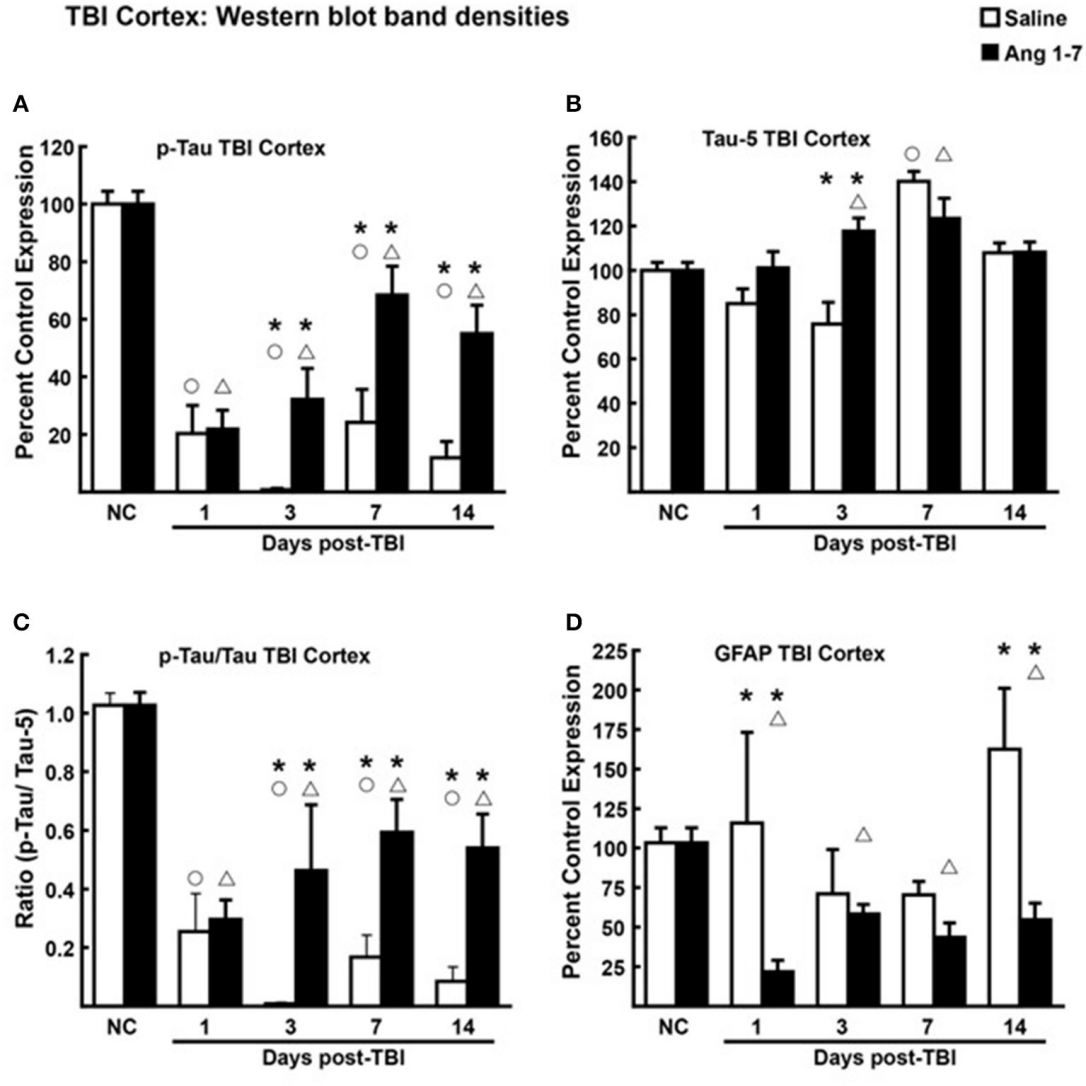
Protein changes in ipsilateral cortex following TBI (*N* = 3–4 mice/group). **(A)** Pooled protein densities of cortex from Western blot studies demonstrate a significant decrease for pTau on days 1, 3, 7, and 14 after a mTBI in both Ang-(1-7) (1 mg/kg, i.p. days 1–5) and saline treated animals. Yet, a significant difference was seen on days 3, 7, and 14 in Ang-(1-7) treated animals as compared to saline with less pTau expression. **(B)** There was a significant increase in Tau on day 7 but not on days 1, 3, or 14, Ang-(1-7) did have a significant effect in increasing overall Tau on day 7 but was no different to saline on the other days. **(C)** The pTau:Tau ratio is expressed relative to 1.0, the pre-TBI control value and demonstrated a significant difference on days 3, 7, and 14 between saline and Ang-(1-7) treatment. **(D)** GFAP was represented as Percent Control Expression for post-TBI days 1, 3, 7, 14. GFAP expression in cortex demonstrated a reverse-U-shaped curve with levels increasing on days 1 and 14 but decreasing on days 3 and 7. Ang-(1-7) demonstrated a significant decrease in GFAP on days 1 and 14 as compared to saline treatment. NC (normal control) is pre-TBI control protein levels standardized to a value of 100. “**°**” indicates a statistically significant difference (*p* < 0.05) in expression level or ratio between the selected post-TBI day and baseline for saline treated animals. “**Δ**” indicates a statistically significant difference (*p* < 0.05) in expression level or ratio between the selected post-TBI day and baseline for Ang-(1-7) treated animals. “*” indicates a statistically significant difference (*p* < 0.05) in expression level or ratio between the Ang-(1-7) and Saline treated groups for the selected post-TBI day (*N* = 4 mice/group).

Tau, a microtubule-associated protein that promotes tubulin assembly, contributes to the stability of microtubules in neuronal axons. Post-translational modification of Tau *via* phosphorylation destabilizes these interactions altering axoplasmic function (Gong et al., 2005). Measurement of Tau and pTau, over the 14 days post-TBI demonstrated statistically significant (*p* < 0.05) changes between Ang-(1-7)-treated mice and saline-treated controls in cortical tissue (Figure 6). For cortical tissue, pooled protein densities from Western blot studies depicted higher expression of pTau [68.4 ± 10.4 Ang-(1-7) vs. 24.2 ± 11.42 Saline, *p* = 0.042] and lower expression of Tau [123.5 ± 9.08 Ang-(1-7) vs. 140.1 ± 4.44 Saline, *p* = 0.13] relative to pre-TBI baseline in the Ang-(1-7) group compared to control on day 7 (Figure 6). This translated to a higher pTau:Tau ratio in Ang-(1-7)-treated mice compared to those treated with saline on day 7 [0.59 ± 0.11 Ang-(1-7) vs. 0.33 ± 0.08 Saline, *p* = 0.01] (Figure 4C). pTau:Tau ratios were greater in the Ang-(1-7) tissue starting on post-TBI day 3 throughout the end of the study (Day 3, *p* = 0.01; Day 7, *p* = 0.011; Day 14, *p* = 0.003).

**Figure 7:**
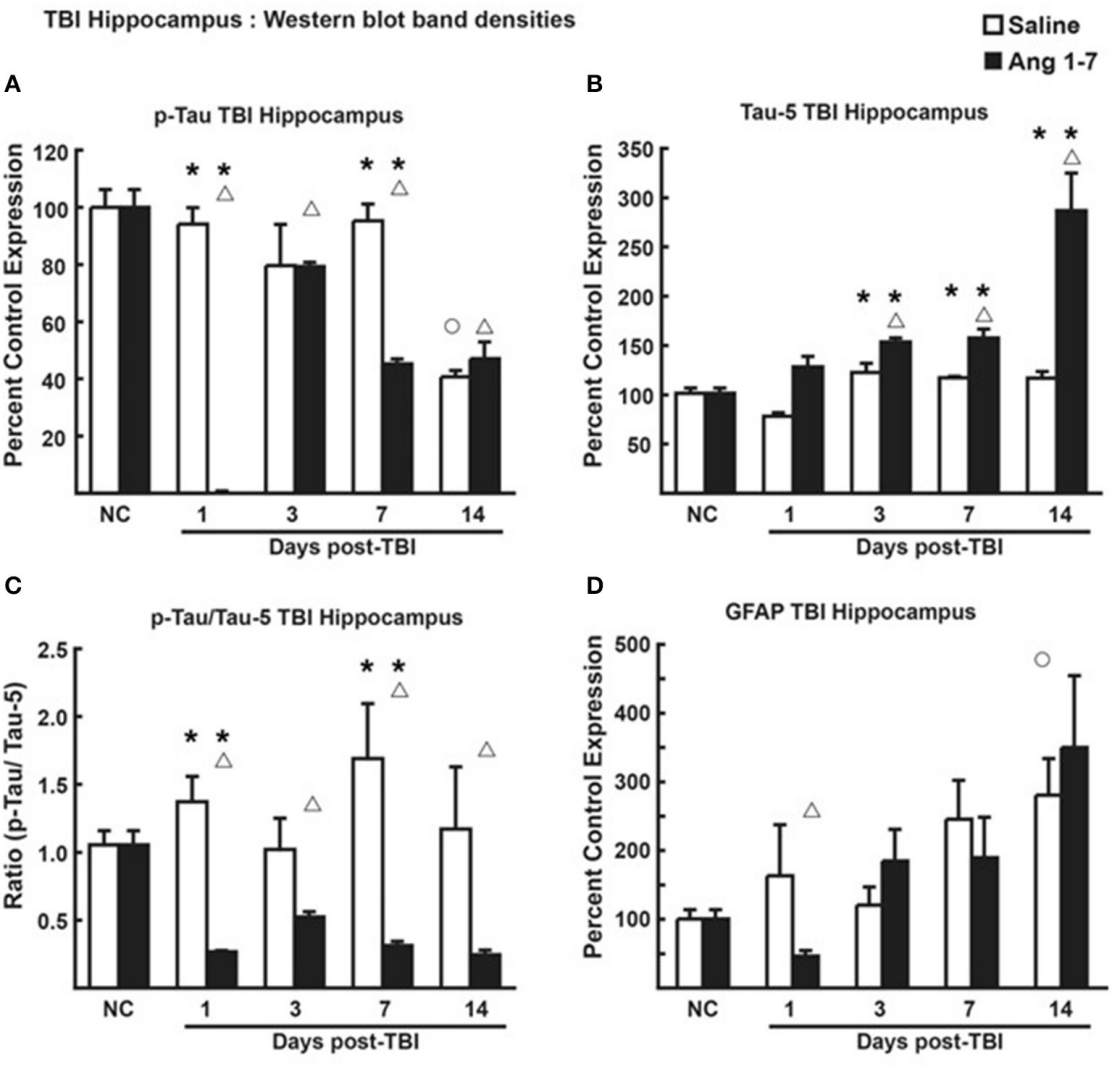
Protein changes in hippocampus following TBI (*n* = 3–4 mice/group). **(A)** Pooled protein densities of left hippocampus from Western blot studies demonstrate a significant decrease for pTau on days 1, 3, 7, and 14 after a mTBI in saline treated animals. Yet, a significant difference was seen on days 1 and 7 in Ang-(1-7) (1 mg/kg, i.p. days 1–5) treated animals as compared to saline with less pTau expression. There were no significant differences on days 3 and 14 between saline and Ang-(1-7) treatments. **(B)** There was a significant increase in Tau on days 3, 7, and 14 after the mTBI in the Ang-(1-7) treatment with day 14 doubling in the amount of hippocampal Tau protein due to Ang-(1-7) treatment. **(C)** The pTau:Tau ratio demonstrated a significant difference on days 1 and 7 with Ang-(1-7) demonstrating a significant decrease in pTau as compared to saline treated animals. A similar trend was seen on days 3 and 14. **(D)** GFAP was represented as Percent Control Expression for post-TBI days 1, 3, 7, and 14. GFAP expression in cortex demonstrated an increase in levels from baseline on days 3, 7, and 14 with no significant difference between treatment groups. NC (normal control) is pre-TBI control protein levels standardized to a value of 100. “**°**” indicates a statistically significant difference (*p* < 0.05) in expression level or ratio between the selected post-TBI day and baseline for saline treated animals. “Δ” indicates a statistically significant difference (*p* < 0.05) in expression level or ratio between the selected post-TBI day and baseline for Ang-(1-7) treated animals. * indicates a statistically significant difference (*p* < 0.05) in expression level or ratio between the Ang-(1-7) and Saline treated groups. *P < 0.05.

In hippocampal tissue, however, pooled protein densities from Western blot studies depicted an inverse relationship from that of the cortex. Pooled protein densities from hippocampal Western blot studies on post-mTBI day 7 showed lower expression of pTau [45.2 ± 1.65 Ang-(1-7) vs. 95.2 ± 5.85 Saline, *p* = 0.0007] (Figure 7A) and higher expression of Tau [157.7 ± 9.11 Ang-(1-7) vs. 117.3 ± 1.34 Saline, *p* = 0.003] (Figure 7B) relative to pre-mTBI baseline in the Ang-(1-7) group as compared to control. This translated to a lower pTau:Tau ratio in Ang-(1-7)-treated mice compared to those treated with saline on day 7 [0.312 ± 0.033 Ang-(1-7) vs. 1.691 ± 0.403 Saline, *p* = 0.02] (Figure 7C). pTau:Tau ratios were consistently lower in the Ang-(1-7) group over the course of the 14 days post-mTBI period (Day 1, *p* = 0.02; Day 3, *p* = 0.0830; Day 7, *p* = 0.02; Day 14, *p* = 0.10).

### Reactive gliosis is reduced in Ang-(1-7) treated mouse cortex

Changes to brain architecture in post-mTBI animals include glial scar formation in damaged regions secondary to astrocyte proliferation. We measured the extent of reactive gliosis using total expression of astrocyte GFAP. In the Ang-(1-7)-treated group, western blot revealed lower relative expression of cortical GFAP compared to pre-mTBI baseline on all days post-mTBI (baseline = 103.3 ± 9.42; Day 1 = 21.7 ± 7.31, *p* = 0.0001; Day 3 = 58.4 ± 5.92, *p* = 0.0007; Day 7 = 43.6 ± 9.08, *p* = 0.007; Day 14 = 42.9 ± 8.22, *p* = 0.0015) (Figure 6D). In saline-treated controls, GFAP expression was not significantly changed from pre-mTBI baseline. Relative expression of cortical GFAP was significantly lower in Ang-(1-7) mice compared to controls by post-mTBI day 14 [42.9 ± 8.22 Ang-(1-7) vs. 162.4 ± 38.66 Saline, *p* = 0.0005] (Figure 6D); this effect was not observed in hippocampal sections (Figure 7D).

### Ang-(1-7) does not significantly change serum levels of proinflammatory mediators post-mTBI

**Figure 8:**
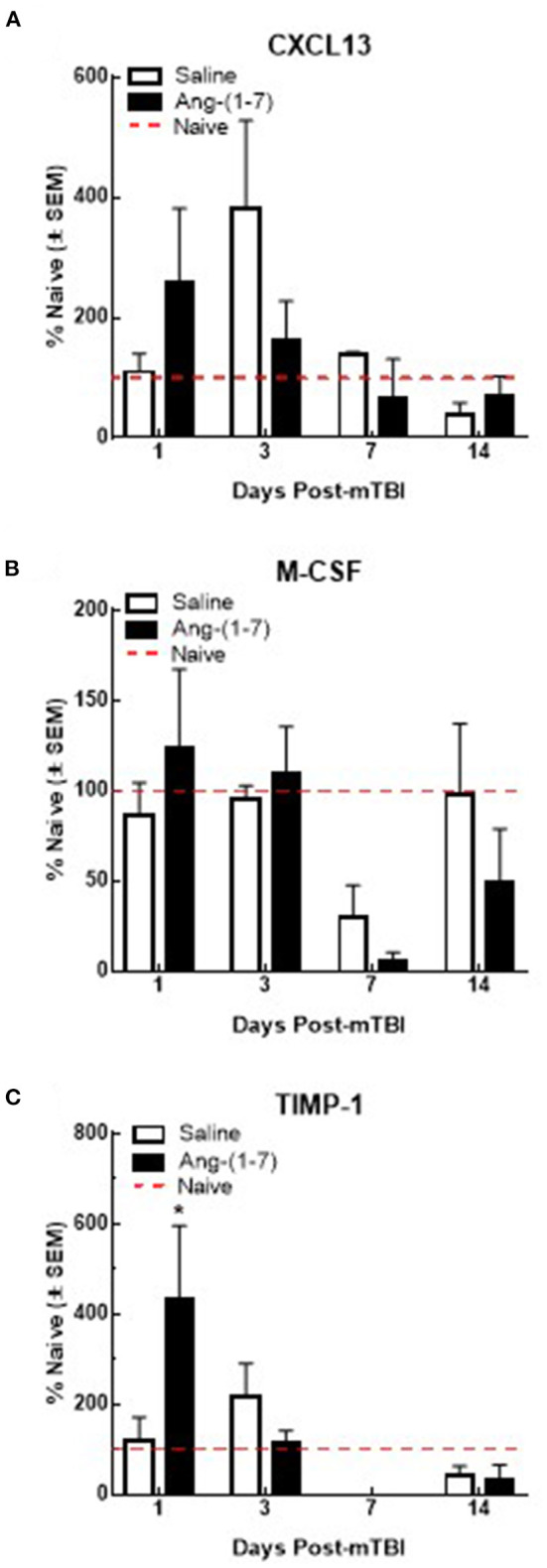
Qualitative changes in serum cytokine levels (*n* = 3–4 mice/group). **(A)** CXCL13 expression increases after 3 days post-mTBI in saline treated animals. Ang-(1-7) (1 mg/kg, i.p.) significantly normalized this mTBI induced CXCL13 increase on D3 (*p < 0.05). No other increases or decreases in CXCL13 expression were observed over the observation period of 14 d. **(B)** M-CSF expression decreases below naïve levels after day 3 post-mTBI in saline and Ang-(1-7) treated animals, at 7 d (*p* < 0.05) with no difference between saline and Ang-(1-7) groups. **(C)** TIMP-1 expression significantly increases in Ang-(1-7) treated animals 1-day post TBI as compared to naïve controls; this increase was not observed in saline treated animals until D3 post TBI 3d post-mTBI; Ang-(1-7) treated animals expressed TIMP-1 at significantly lower levels than saline treated animals (*p* = 0.03).

Previous studies suggest that circulating levels of proinflammatory cytokines are increased after mTBI (Kelley et al., 2007; Rowe et al., 2016). To assess the proinflammatory response in Ang-(1-7)-treated mTBI mice, serum samples were analyzed for 40 inflammatory cytokines on days 1, 3, 7, and 14 post-mTBI. Of the cytokines assessed, levels of six were detected after mTBI: CXCL13, C5/C5a, sICAM-1, M-CSF, SDF-1, and TIMP-1. mTBI increased serum CXCL13 levels on day 3 post-injury in saline-treated mice (382.5 ± 145.1% over naïve); this was reduced by Ang-(1-7) intervention, albeit not significantly (162.7 ± 145.1%; Figure 8A). M-CSF expression in serum was decreased below naïve levels post-mTBI by day 7 in both saline (29.5 ± 18.2%) and Ang-(1-7) groups (5.3 ± 5.3%; Figure 8B); no statistical difference was observed between treatment groups. TIMP1 showed bidirectional fluctuations after mTBI. On day 1 post-mTBI, serum from Ang-(1-7)-treated mice showed an increased in TIMP-1 relative to naïve mice (432.9 ± 163.8% over naïve); levels of TIMP in saline-treated mice were similar to controls (121.2 ± 50.9%). By day 7, TIMP1 was no longer detected in serum samples from mTBI mice (Figure 8C). Whilst C5/C5a, sICAM-1, and SDF-1 were detected in serum after mTBI, none were changed following any intervention.

## Discussion

TBI results in millions of emergency department visits, hospitalizations, and deaths each year while many more go undiagnosed and untreated by primary, urgent, and specialty care clinicians (Vos et al., 2002; Menon et al., 2010; Traumatic Brain Injury/Concussion | Concussion | Traumatic Brain Injury | CDC Injury Center., 2021). mTBI is common and may suppress cognitive function in the long-term, yet there exist no proven therapies to mitigate these potential negative outcomes. Here, studies examined the therapeutic effect of Ang-(1-7) on secondary injury observed in a murine model of mTBI in a closed skull, single injury model. Ang-(1-7) inhibited the cognitive deficits of mTBI, reduced neuronal loss, and reduced levels of phosphorylated Tau in the hippocampus during the secondary injury phase of mTBI.

Ang-(1-7) maintained cognition on days 1–3 following mTBI but lost efficacy by days 5–8, suggesting tolerance; however, discontinuation of Ang-(1-7) after day 5 resulted in a rebound effect, suggesting that early treatment may inhibit long-term deficits of mTBI. Importantly, the beneficial effects of Ang-(1-7) on post-mTBI cognition were shown *not* to be dose-dependent, as no significant differences in NOR were observed between the 0.1 and 0.3 mg/kg Ang-(1-7) groups. Finally, in order to bolster the argument that the role of Ang-(1-7) in post-injury neuroprotection is MASR-mediated, pre-treatment with MASR antagonist A779 was employed alongside Ang-(1-7) administration in a group of mice again subject to mTBI. A779 pre-treatment not only nullified the beneficial effects of exogenous Ang-(1-7) (which should render the treatment no better or worse than saline) but precipitated significant *reduction* in cognitive function relative to saline on days 2 and 4 following mTBI. Not only does this support the hypothesis that the MASR axis serves an important function in the attenuation of secondary injury post-mTBI, but also brings into question the role of endogenous Ang-(1-7) in the natural mammalian defense against the sequelae of neurotrauma. Further investigation into the deleterious effects of MASR antagonism in the setting of trauma is therefore warranted. A similar finding was reported by Janatpour et al. (2019) using an open-skull, single cortical injury model, wherein Ang-(1-7) attenuated motor deficits at 3 days post-injury and improved performance in the Morris water maze at 28 days post-injury.

This long-term effect of Ang-(1-7) treatment was further supported by histopathology demonstrating maintenance and steady improvement of tissue architecture and pyramidal neuron counts, respectively, over 7–14 days in both the cortex and hippocampus ipsilateral to the site of injury. Interestingly, the contralateral hippocampus demonstrated neuronal loss that was also attenuated by the administration of Ang-(1-7), suggesting that there may be widespread effects in the CNS post-mTBI, and that systemic administration of the peptide may reach all areas of the CNS vulnerable to damage. The reduction of mTBI-induced GFAP expression in the cortex on days 1 and 14 by Ang-(1-7) suggests that Mas receptor activation on glial cells may reduce overall CNS inflammation and gliosis (Janatpour et al., 2019). Yet, the lack of significant effect on days 3 and 7 are likely due to the once-daily administration paradigm and the absence of dose accumulation required to overcome the cortical inflammation and damage generated by mTBI. Here in this study, we did not examine microglial response, as microglia are not used in the clinicopathological diagnostic setting to judge cortical injury; however, astrogliosis and neuronal injury/death are commonly used.

Increased pTau has been linked to several CNS diseases in humans, including mTBI that results in cognitive impairment over time (Morris, 2011; Shahim et al., 2014). The mTBI-induced increase in the ratio of pTau to Tau in the hippocampus was significantly reduced by Ang-(1-7) treatment, supporting a neuroprotective role for Ang-(1-7). While these are the first reports of Ang-(1-7) effects on pTau, further studies are required to determine whether increased dosing and/or sustained administration of Ang-(1-7) proves more beneficial. Thus, mTBI significantly damages the CNS based on the histologic and behavioral metrics observed, and this damage is mitigated and reversed by Ang-(1-7).

Corresponding to the cognitive changes, significant differences in cytokine levels between the treatment and control groups existed. Specifically, brain levels of CXCL-13 and Timp-1 were increased on day 1. However, typical proinflammatory mediators such as IL-6, IL-1β, and TNFα were not detected above baseline levels from day 1 to 14 suggesting that the conditions favored a mild injury. Recent studies indicate that MasR activation in the CNS decreases levels of reactive oxygen species (Zhou et al., 2018). Recent studies using cardiomyocytes demonstrate that Ang-(1-7) acting at MASR protects from PI3/Akt-induced apoptosis (Yang et al., 2018), supporting an Ang-(1-7)/MASR cellular protective effect following severe stress. Such studies with the current mTBI model must be explored.

Critically, Ang-(1-7) induced a statistically significant decrease in pTau and functional Tau in the hippocampus compared to control animals 1–14 days post-mTBI, suggesting a neuroprotective role. Previous studies have demonstrated the neuroprotective role of Ang-(1-7) in stroke, as it attenuates the inflammatory response generated during the “second-hit” phase of TBI by increasing vasodilation and cerebral blood flow, decreasing oxidative stress, and reducing pro-inflammatory gene expression (Zheng et al., 2014). Likewise, we believe the RAAS metabolite employs a similar mechanism to prevent long-term inflammatory damage following mTBI.

Similar studies in other areas of cognitive impairment have investigated Ang-(1-7) including models of Alzheimer's disease in which Ang-(1-7) demonstrate therapeutic potential by attenuating Aβ42-induced changes linked to behavioral and molecular observation including memory impairment and. attenuation of tau hyperphosphorylation (pTau) within the hippocampus (Chen et al., 2017). Ang-(1-7) was shown to be cerebroprotective in aging animals by attenuating the loss of endothelial function of cerebral arteries that often occurs with aging and correlated with ACE2 deficiency (Peña-Silva et al., 2012). Ang-(1-7) has a potential therapeutic strategy for delayed cerebral ischemia in subarachnoid hemorrhage and reduce brain damage. Cognitive impairment in a murine model of congestive heart failure (CHF) exhibited both spatial memory and object recognition dysfunction, while systemic administration of Ang-(1-7) improved spatial memory in CHF mice and attenuate a cognitive decline as compared with shams (Hay et al., 2017). Here, in agreement with prior studies, Ang-(1-7) is shown to be structurally and functionally neuroprotective in a closed-skull, controlled cortical impact model of mTBI, supporting its potential in clinical utility for the treatment of traumatic brain injury.

## Data availability statement

The original contributions presented in the study are included in the article/Supplementary material, further inquiries can be directed to the corresponding author.

## Ethics statement

The animal study was reviewed and approved by the University of Arizona Institutional Animal Care and Use Committee.

## Author contributions

All authors contributed to study design, literature search, data collection, analysis and interpretation, writing, and critical revision of manuscript drafts.

## Funding

This work was supported by Grants from the U.S. Army Research Laboratory and Defense (W911NF-15-1-0093; BJ), Arizona Biomedical Research Contract (ADHS18-198853), and UArizon's Comprehensive Pain and Addiction Center and the NIH/NIDA (P30 DA051355).

## Conflict of interest

The authors declare that the research was conducted in the absence of any commercial or financial relationships that could be construed as a potential conflict of interest.

## Publisher's note

All claims expressed in this article are solely those of the authors and do not necessarily represent those of their affiliated organizations, or those of the publisher, the editors and the reviewers. Any product that may be evaluated in this article, or claim that may be made by its manufacturer, is not guaranteed or endorsed by the publisher.

## Author disclaimer

The authors are solely responsible for this content, which does not necessarily represent the official views, policies, or opinions of the DOD, National Institutes of Health, or the University of Arizona.

## Supplementary material

Supplementary Figure 1 Ang-(1-7)-mediated improvement in cognitive function does not exhibit dose dependence between the 0.1 and 0.3 mg/kg groups. Demonstrates that 0.1 and 0.3 mg/kg groups mediated improvement in cognitive function and there is significant difference (^*^*p* < 0.05 on Day 2, 0.1 and 0.3 mg/kg groups vs. Saline). *N* = 8–12 mice/group.

## References

[B1] Albayram O. Kondo A. Mannix R. Smith C. Tsai C. Y. Li C.. (2017). Cis P-Tau is induced in clinical and preclinical brain injury and contributes to post-injury sequelae. Nat. Commun. 8, 1000. 10.1038/s41467-017-01068-4 29042562 PMC5645414

[B2] Arenth P. M. Russell K. C. Scanlon J. M. Kessler L. J. Ricker J. H. (2014). Corpus callosum integrity and neuropsychological performance after traumatic brain injury: a diffusion tensor imaging study. J. Head Trauma Rehabil. 29, E1–E10. 10.1097/HTR.0b013e318289ede5 23558829 PMC4918513

[B3] Cassidy J. D. Carroll L. Peloso P. Borg J. Von Holst H. Holm L.. (2004). Incidence, risk factors and prevention of mild traumatic brain injury: results of the WHO collaborating centre task force on mild traumatic brain injury. J. Rehabil. Med. Suppl. 43, 28–60. 10.1080/16501960410023732 15083870

[B4] Chen J. L. Zhang D. L. Sun Y. Zhao Y. X. Zhao K. X. Pu D.. (2017). Angiotensin-(1–7) administration attenuates Alzheimer's disease-like neuropathology in rats with streptozotocin-induced diabetes *via* mas receptor activation. Neuroscience 346, 267–277. 10.1016/j.neuroscience.2017.01.027 28147245

[B5] Forte B. L. Slosky L. M. Zhang H. Arnold M. R. Staatz W. D. Hay M.. (2016). Angiotensin-(1-7)/mas receptor as an antinociceptive agent in cancer-induced bone pain. Pain 157, 690. 10.1097/j.pain.0000000000000690 27541850 PMC5108669

[B6] Frati A. Cerretani D. Fiaschi A. I. Frati P. Gatto V. La Russa R.. (2017). Diffuse axonal injury and oxidative stress: a comprehensive review. Int. J. Mol. Sci. 18, 2709–21. 10.3390/ijms18122600 29207487 PMC5751203

[B7] Gong C. X. Liu F. Grundke-Iqbal I. Iqbal K. (2005). Post-translational modifications of tau protein in Alzheimer's disease. J. Neural Trans. 112, 813–838. 10.1007/s00702-004-0221-0 15517432

[B8] Hay M. Vanderah T. W. Samareh-Jahani F. Constantopoulos E. Uprety A. R. Barnes C. A.. (2017). Cognitive impairment in heart failure: a protective role for angiotensin-(1-7). Behav. Neurosci. 131, 99–114. 10.1037/bne0000182 28054808 PMC6456812

[B9] Jackson T. R. Blair L. A. C. Marshall J. Goedert M. Hanley M. R. (1988). The mas oncogene encodes an angiotensin receptor. Nature 335, 437–440. 10.1038/335437a0 3419518

[B10] Janatpour Z. C. Korotcov A. Bosomtwi A. Dardzinski B. J. Symes A. J. (2019). Subcutaneous administration of angiotensin-(1-7) improves recovery after traumatic brain injury in mice. J. Neurotr. 36, 3115–3131. 10.1089/neu.2019.6376 31037999

[B11] Jiang T. Gao L. Lu J. Zhang Y. D. (2013). ACE2-ang-(1-7)-mas axis in brain: a potential target for prevention and treatment of ischemic stroke. Curr. Neuropharmacol. 11, 209–217. 10.2174/1570159X11311020007 23997755 PMC3637674

[B12] Johnson V. E. Stewart J. E. Begbie F. D. Trojanowski J. Q. Smith D. H. Stewart W. (2013). Inflammation and white matter degeneration persist for years after a single traumatic brain injury. Brain 136, 28–42. 10.1093/brain/aws322 23365092 PMC3562078

[B13] Joseph B. Pandit V. Zangbar B. Kulvatunyou N. Khalil M. Tang A.. (2015). Secondary brain injury in trauma patients: the effects of remote ischemic conditioning. J. Trauma Acute Care Surg. 78, 698–705. 10.1097/TA.0000000000000584 25742251

[B14] Kelley B. J. Lifshitz J. Povlishock J. T. (2007). Neuroinflammatory responses after experimental diffuse traumatic brain injury. J. Neuropathol. Exp. Neurol. 66, 989–1001. 10.1097/NEN.0b013e3181588245 17984681

[B15] Kulbe J. R. Hall E. D. (2017). Chronic traumatic encephalopathy-integration of canonical traumatic brain injury secondary injury mechanisms with tau pathology. Prog. Neurobiol. 158, 15–44. 10.1016/j.pneurobio.2017.08.003 28851546 PMC5671903

[B16] Mehta P. K. Griendling K. K. (2007). Angiotensin II cell signaling: Physiological and pathological effects in the cardiovascular system. Am. J. Physiol. 292, 82–97. 10.1152/ajpcell.00287.2006 16870827

[B17] Menon D. K. Schwab K. Wright D. W. Maas A. I. (2010). Position statement: definition of traumatic brain injury. Arch. Phys. Med. Rehabil. 91, 1637–1640. 10.1016/j.apmr.2010.05.017 21044706

[B18] Morris M. (2011). The main faces of tau. Neuron 29, 997–1003. 10.1016/j.biotechadv.2011.08.021 21555069 PMC3319390

[B19] Passos-Silva D. G. Verano-Braga T. Santos R. A. (2013). Angiotensin-(1-7): beyond the cardio-renal actions. Clin. Sci. 124, 443–456. 10.1042/CS20120461 23249272

[B20] Peña-Silva R. A. Chu Y. Miller J. D. Mitchell I. J. Penninger J. M. Faraci F. M.. (2012). Impact of ACE2 deficiency and oxidative stress on cerebrovascular function with aging. Stroke 43, 3358–3363. 10.1161/STROKEAHA.112.667063 23160880 PMC3529166

[B21] Pörsti I. Bara A. T. Busse R. Hecker M. (1994). Release of nitric oxide by angiotensin-(1–7) from porcine coronary endothelium: implications for a novel angiotensin receptor. Br. J. Pharmacol. 111, 652–654. 10.1111/j.1476-5381.1994.tb14787.x 8019744 PMC1910086

[B22] Rowe R. K. Ellis G. I. Harrison J. L. Bachstetter A. D. Corder G. F. Van Eldik L. J.. (2016). Diffuse traumatic brain injury induces prolonged immune dysregulation and potentiates hyperalgesia following a peripheral immune challenge. Mol. Pain 12, 1–12. 10.1177/1744806916647055 27178244 PMC4955995

[B23] Sandweiss A. J. Azim A. Ibraheem K. Largent-Milnes T. M. Rhee P. Vanderah T. W.. (2017). Remote ischemic conditioning preserves cognition and motor coordination in a mouse model of traumatic brain injury. J. Trauma Acute Care Surg. 83, 1074–1081. 10.1097/TA.0000000000001626 28609381

[B24] Santos R. A. S. Simoes A. C. Maric C. Silva D. M. R. Machado R. P. De Buhr I.. (2003). G protein-coupled receptor mas. Proc. Natl. Acad. U. S. A. 100, 8258–8263. 10.1073/pnas.1432869100 12829792 PMC166216

[B25] Shahim P. Tegner Y. Wilson D. H. Randall J. Skillbäck T. Pazooki D.. (2014). blood biomarkers for brain injury in concussed professional ice hockey players. JAMA Neurol. 71, 684–692. 10.1001/jamaneurol.2014.367 24627036

[B26] Silvers J. M. Harrod S. B. Mactutus C. F. Booze R. M. (2007). Automation of the novel object recognition task for use in adolescent rats. J. Neurosci. Met. 166, 99–103. 10.1016/j.jneumeth.2007.06.032 17719091 PMC3184886

[B27] Terrio H. Brenner L. A. Ivins B. J. Cho J. M. Helmick K. Schwab K.. (2009). Traumatic brain injury screening: preliminary findings in a US army brigade combat team. J. Head Trauma Rehabil. 24, 14–23. 10.1097/HTR.0b013e31819581d8 19158592

[B28] Traumatic Brain Injury/Concussion | Concussion | Traumatic Brain Injury | CDC Injury Center. (2021). Cdc. Available online at: https://www.cdc.gov/traumaticbraininjury/index.html

[B29] Vos P. E. Battistin L. Birbamer G. Gerstenbrand F. Potapov A. Prevec T.. (2002). EFNS guideline on mild traumatic brain injury: report of an EFNS task force. European Journal of Neurology 9 (3):207–219. 10.1046/j.1468-1331.2002.00407.x 11985628

[B30] Yang Y.-Y. Sun X.-T. Li Z.-X. Chen W.-Y. Wang X. Liang M.-L.. (2018). Protective effect of angiotensin-(1-7) against hyperglycaemia-induced injury in H9c2 cardiomyoblast cells *via* the PI3K?Akt signaling pathway. Int. J. Mol. Med. 41, 1283–1292. 10.3892/ijmm.2017.3322 29286068 PMC5819934

[B31] Zheng J. L. Li G. Z. Chen S. Z. Wang J. J. Olson J. E. Xia H. J.. (2014). Angiotensin converting enzyme 2/Ang-(1-7)/mas axis protects brain from ischemic injury with a tendency of age-dependence. CNS Neurosci. Ther. 20, 452–459. 10.1111/cns.12233 24581232 PMC4840841

[B32] Zhou Y. Li M. Zhu D. L. Jiang T. Gao Q. Tang X. H.. (2018). Neuroprotective effect of angiotensin-(1–7) against rotenone-induced oxidative damage in CATH.a neurons. Toxicol. Vitro 50, 373–382. 10.1016/j.tiv.2018.04.005 29665408

