# Supplementary figures and images for: Angiotensin-(1-7) improves cognitive function and reduces inflammation in mice following mild traumatic brain injury
Source: Front Behav Neurosci. 2022 Aug 4;16:903980. doi: 10.3389/fnbeh.2022.903980 (PMC9386567; doi:10.3389/fnbeh.2022.903980)

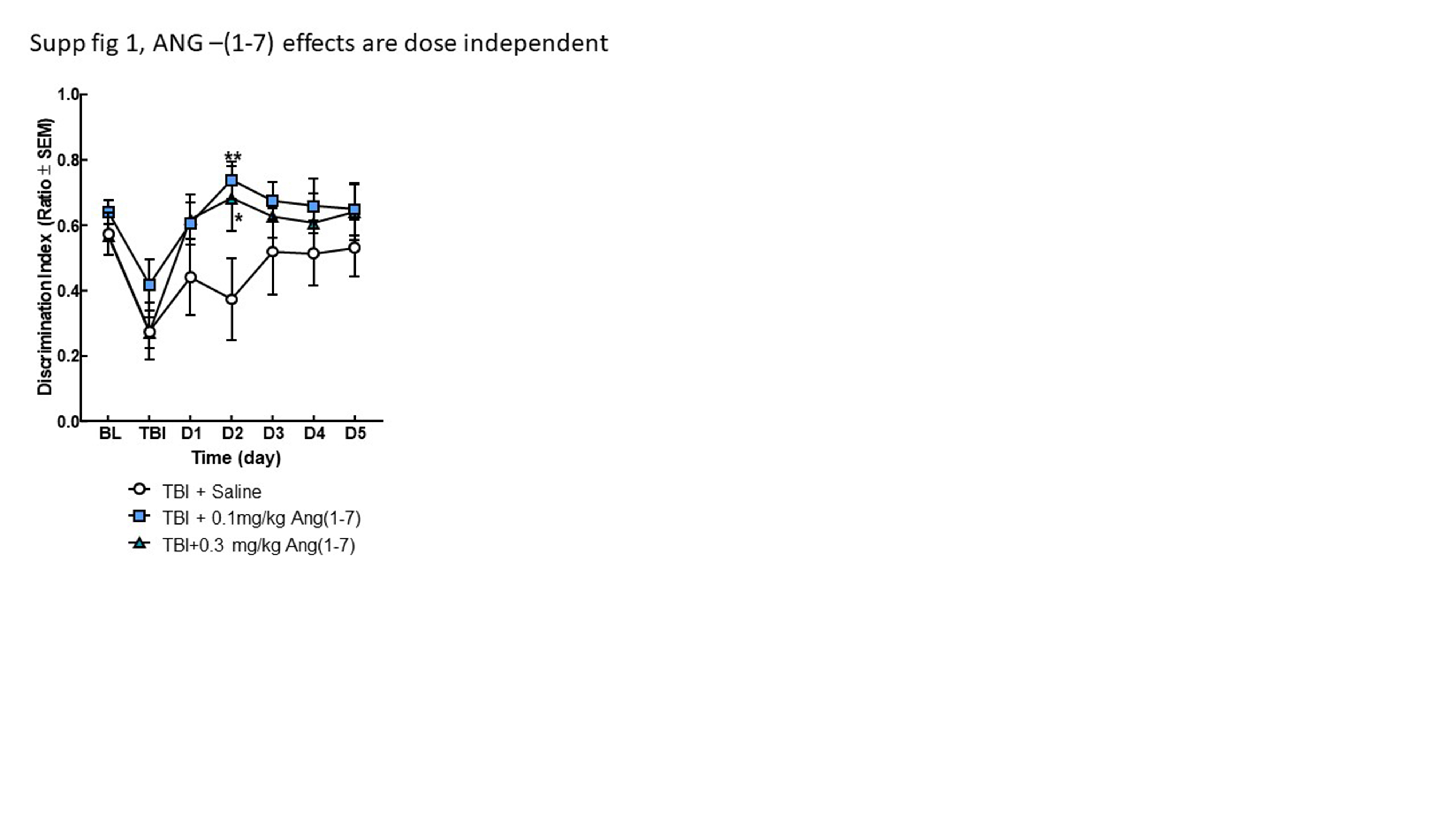

Supplement: Supplementary Figure 1 — Ang-(1-7)-mediated improvement in cognitive function does not exhibit dose dependence between the 0.1 and 0.3 mg/kg groups. Demonstrates that 0.1 and 0.3 mg/kg groups mediated improvement in cognitive function and there is significant difference (*p < 0.05 on Day 2, 0.1 and 0.3 mg/kg groups vs. Saline). N = 8–12 mice/group. [file Image_1.JPEG]
